# Diagnosis of benign and malignant nodules with a radiomics model integrating features from nodules and mammary regions on DCE-MRI

**DOI:** 10.3389/fonc.2024.1307907

**Published:** 2024-02-21

**Authors:** Wei Fan, Wei Sun, Ming Ze Xu, Jing Jing Pan, Feng Yuan Man

**Affiliations:** ^1^ Department of Radiology, Rocket Force Characteristic Medical Center of the Chinese People's Liberation Army, Beijing, China; ^2^ Department of Interventional Therapy, National Cancer Center/National Clinical Research Center for Cancer/Cancer Hospital, Chinese Academy of Medical Sciences and Peking Union Medical College, Beijing, China; ^3^ Postgraduate Training Base of Jinzhou Medical University, Rocket Force Characteristic Medical Center of the Chinese People’s Liberation Army, Beijing, China

**Keywords:** breast cancer, radiomics, DCE-MRI, nodule region, mammary region

## Abstract

**Objectives:**

To establish a radiomics model for distinguishing between the benign and malignant mammary gland nodules via combining the features from nodule and mammary regions on DCE-MRI

**Methods:**

In this retrospective study, a total of 103 cases with mammary gland nodules (malignant/benign = 80/23) underwent DCE-MRI, and was confirmed by biopsy pathology. Features were extracted from both nodule region and mammary region on DCE-MRI. Three SVM classifiers were built for diagnosis of benign and malignant nodules as follows: the model with the features only from nodule region (N model), with the features only from mammary region (M model) and the model combining the features from nodule region and mammary region (NM model). The performance of models was evaluated with the area under the curve of receiver operating characteristic (AUC).

**Results:**

One radiomic features is selected from nodule region and 3 radiomic features is selected from mammary region. Compared with N or M model, NM model exhibited the best performance with an AUC of 0.756.

**Conclusions:**

Compared with the model only using the features from nodule or mammary region, the radiomics-based model combining the features from nodule and mammary region outperformed in the diagnosis of benign and malignant nodules.

## Introduction

Breast cancer is the most prevalent cancer and the second leading of the death caused by cancer among women overall (1). Early diagnosis and treatment contributed to decreasing the death rate and improving the prognosis of patients with breast cancer (2). Pathological examination is an invasive examination for clinical diagnosis of breast cancer. Breast MRI and especially dynamic contrast enhanced MRI (DCE-MRI) can provide morphological and anatomical information due to its advantage on soft tissue imaging with high contrast. Compared with mammography and ultrasound, DCE-MRI exhibited a superior sensitivity and negative likelihood ratios with higher pretest probabilities to rule out malignancy (3). Previous study had proved the potential of DCE-MRI in the diagnosis prognosis and treatment response evaluation of breast cancer (4, 5).

Traditional recognition of benign and malignant lesions by radiologists relied on the subjective evaluation according to the morphological features and enhanced time course. In addition, the varying tissue contrast at different time points made it a challenge for computer aided image analysis. The advances in computer-aided image analysis contributed to non-invasive evaluation for breast cancer (6, 7). The radiomics based methods can extract high-dimensional and imperceptible features and dig out intratumor heterogeneity that has significant prognostic value. The performance of radiomics-based methods had been validated in diagnosis, staging, determining molecular subtype and predicting lymph node metastasis and disease-free survival of breast cancer (8–10).

Recently, the inadequacy of the features only from intra-tumoral region attracted the attention. To improve the performance of methods, some studies investigated the value of the features from peritumoral region (11, 12). Considering the increased blood supply in the patients with breast cancer, we aimed to combine the features from intra- nodules and mammary region for precise diagnosis of benign and malignant breast lesions in the present study.

## Materials and methods

### Patient enrollment

This retrospective study included patients with breast nodules in March 2018 from to April 2022, and was approved by a local review committee. Each patient offered informed consent before enrollment. The patients were enrolled according to following criteria: 1) female; 2) no treatment for breast surgery and puncture; 3) no history of other malignancies. All the patients were diagnosed by histopathology. The histological grading and subtypes percentage of patients in **Table 1**. Finally, 23 patients with benign nodules and 80 with malignant nodules were eligible for the study.

**Table 1:** The histological grading and subtypes percentage of patients.

Subtypes	Number (percentage)
Benign	23 (22.33%)
Intraductal papilloma	6 (26.09%)
Adenosis of breast	7 (30.43%)
Fibroadenoma of breast	10 (43.48%)
Malignant	80 (77.67%)
Carcinoma in situ	11 (13.75%)
1^g^	0 (0%)
2^g^	10 (90.91%)
3^g^	1 (9.09%)
Invasive carcinoma	69 (86.25%)
1^g^	0 (0%)
2^g^	49 (71.01%)
3^g^	20 (28.99%)

g: histological grading. Histological grading is based on the latest World Health Organization (WHO) classification of breast cancer: degree of ductal formation, nuclear pleomorphism, and ability to divide the nucleus, each item is divided into three points according to different levels. 3-5 points: grade 1, 6-7 points: grade 2, 8-9 points: grade 3.

### MRI protocol

All patients underwent MR scan on a 3.0 Tesla scanner (Magnetom Skyra, Siemens, Germany). A standardized protocol containing three MRI sequences were applied in this study: 1) Axial T2-weighted TIRM with TR/TE = 4000/53 ms, matrix size = 256 × 256, pixel size = 1.4 × 1.4 mm^2^, slice thickness = 4.0 mm, slice spacing = 0.8 mm; 2) axial pre-contrast T1-weighted FLASH with TR/TE = 6.00/2.46 ms, matrix size = 370 × 448, pixel size = 0.8 × 0.8 mm^2^, slice thickness = 1.6 mm, slice spacing = 0.3 mm, flip angle = 15°; 3) axial DCE T1-weighted VIBE with TR/TE = 5.08/1.68 ms, matrix size = 280 × 352, pixel size = 1.0 × 1.0 mm^2^, slice thickness = 4.0 mm, slice spacing = 0.8 mm, flip angle = 15°. Each dynamic frame repeated thirty-five times without time gap, and the total of dynamic scan was around 15 minutes. The gadolinium-based contrast agent was injected using a power injector with a patient-weight-independent dose of 0.2 mmol/kg and a rate of 2.0 ml/s. This was followed by a saline flush.

### Feature extraction and model building

**Figure 1:**
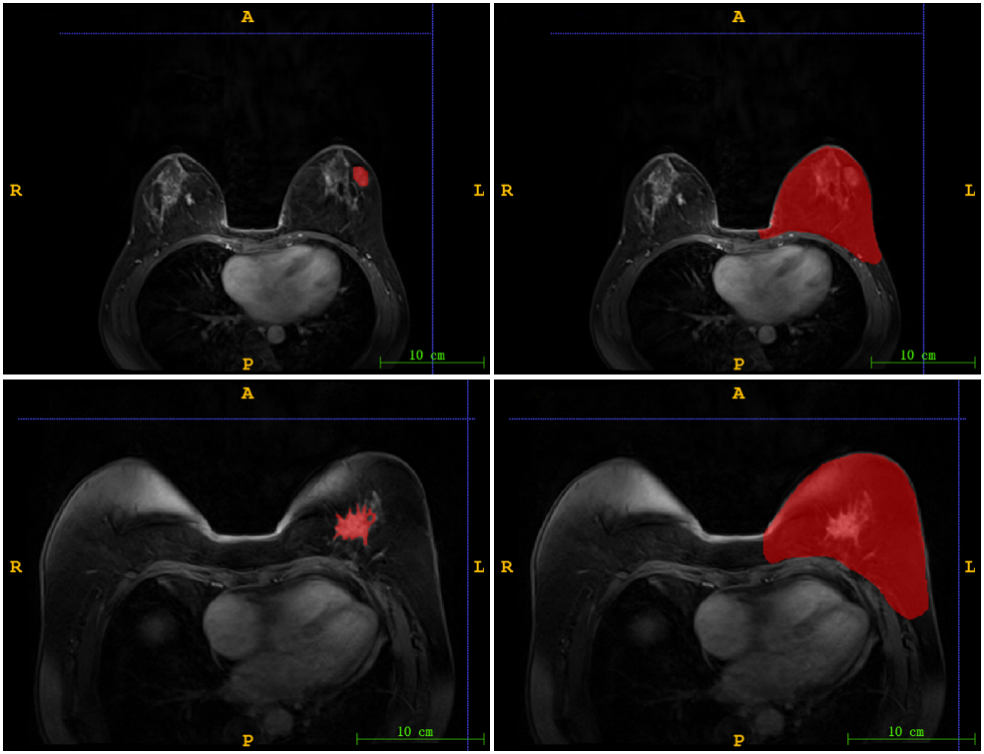
The illustration of two ROIs. Left column: nodule region (N-ROI), right column: mammary region (M-ROI), the first row: benign, the second row: malignant.

Using the ITK-SNAP software (www.itksnap.org, version 4.0.2), we delineated two regions of interest (ROI): nodule region and mammary region. One radiologist with over 10 years` experience in interpreting mammary MR images delineated the boundary of each lesion as well as the whole breast on DCE images. **Figure 1** illustrated two ROIs from two patients. A total of 72 cases were randomly selected as the training data set (malignant/benign = 56/16), and another 31 cases as the independent testing data set (malignant/benign = 24/7).

The intensity of images was scaled to [0, 255] with linear normalization. A total of 851 3D radiomics features (first order features, shaped-based features, GLCM, GLRLM, GLSZM, GLDM, NGTDM) were extracted with a bin width of 16 on original and wavelet images. To remove the unbalance between positive and negative samples, up-sampling was conducted in the present study. Using up-sampling data on the training set refers to augmenting minority samples with random duplication to match the same sample size as the majority samples (malignant/benign = 56/56). Next, a z-score normalization was applied on the feature matrix. Since the dimension of feature space was high, the similarity of each feature pair was compared and one of them was randomly removed if Pearson correlation coefficient (PCC) of the feature pair was larger than 0.90. PCC can efficiently and intuitively reduce dimensionality in large-scale datasets. After this process, the dimension of the feature space was reduced and each feature was independent to each other.

**Figure 2:**
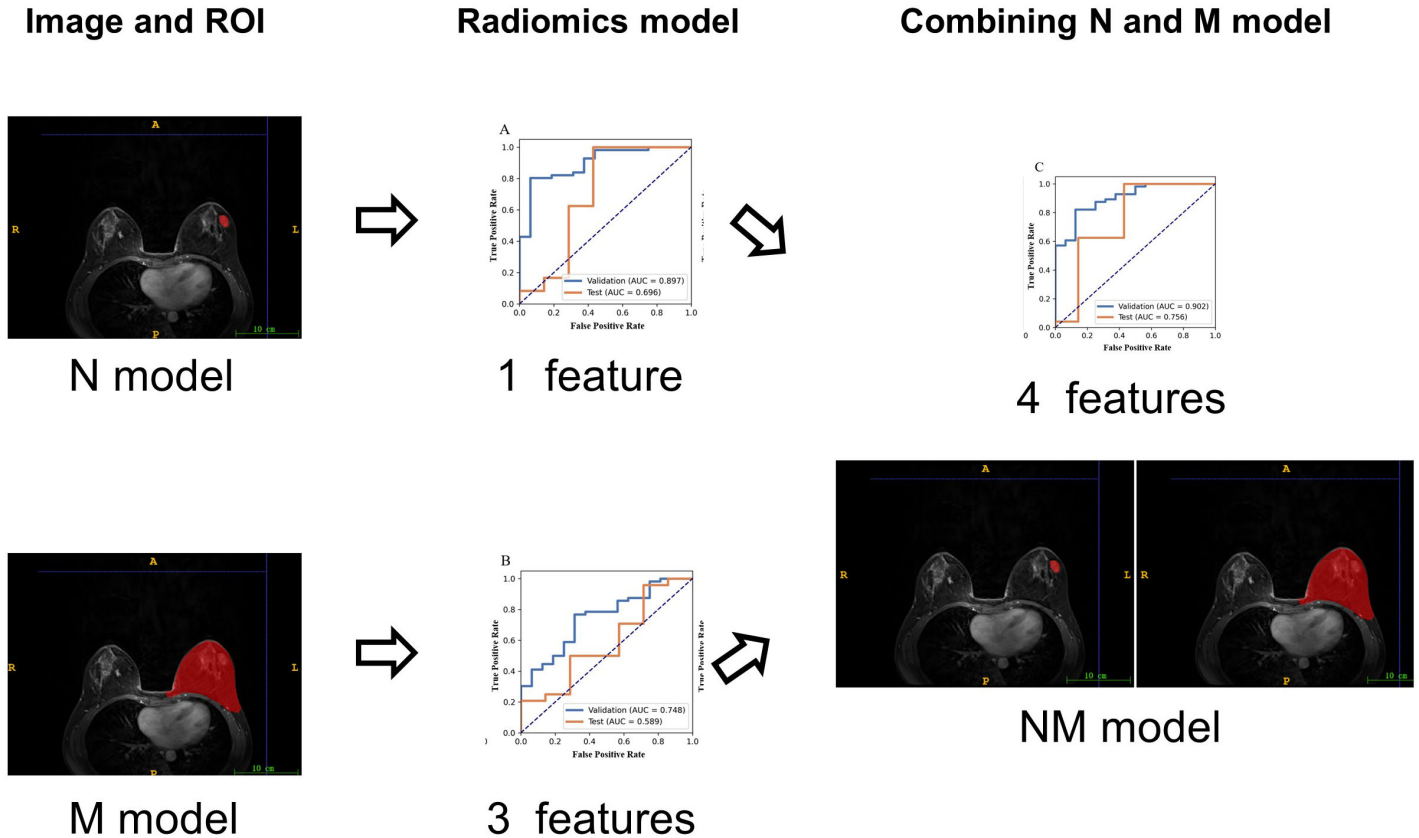
The workflow of radiomics analysis.

Before building the prediction model, the analysis of variance (ANOVA) was used to select features. ANOVA is a quantitative analysis method characterized by strong comparability and high precision. It is suitable for large data samples and can effectively identify variables that are statistically significant. F-value was calculated to evaluate the relationship between features and the label. We sorted features according to the corresponding F-value and selected specific number of features. Then, logistic regression with LASSO was applied for screening of features to reduce the dimension of the feature space. The use of LASSO effectively reduces model complexity and enhances generalization performance. To determine the hyper-parameter of model, cross validation with 5-fold on the training dataset were applied. Finally, classifier was built using support vector machine. The hyper-parameters were set according to the model performance on the validation data set. The workflow of the radiomics models (**Figure 2**). All the processes above were conducted using FAE (https://github.com/salan668/FAE).

### Statistical analysis

The performance of the models was evaluated using receiver operating characteristic (ROC) curve analysis. The area under the curve (AUC) was calculated for quantification. The accuracy, sensitivity, specificity, positive predictive value (PPV), and negative predictive value (NPV) were also calculated at a cutoff value that maximized the value of the Youden index. The 95% confidence interval was also estimated by bootstrap with 1000 samples. All statistical analyses were implemented on Python (3.7.6).

## Results

**Figure 3:**
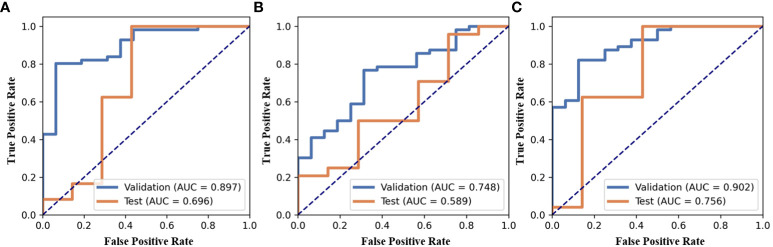
ROC of three models, **(A)** the model with the features only from nodule region (N model), **(B)** the model with the features only from mammary region (M model), **(C)** the model combining the features from nodule region and mammary region (NM model).

In the present study, three models were built for diagnosis of benign and malignant nodules as follows: 1) the model with the features only from nodule region (N model); 2) the model with the features only from mammary region (M model); 3) the model combining the features from nodule region and mammary region (NM model). In the N and M models, there are 851 features each. Finally, after feature selection, N model adds 1 feature, M model adds 3 features and NM model adds 4 features. The extracted features are shown in **Table 2**. Compared with N or M model, NM model exhibited the best performance with an AUC of 0.756 (**Table 3**, **Figure 3**).

**Table 2:** The selected features from two ROIs .

ROI	Feature Name
nodule region (N-ROI)	N_wavelet-LHL_glcm_MCC
mammary region (M-ROI)	M_wavelet-LLH_firstorder_Maximum
	M_wavelet-HHH_firstorder_Mean
	M_wavelet-HHH_firstorder_Mean

**Table 3:** The performance of SVM classifiers with features from different ROI.

Model	AUC	95% CI	Cutoff	MCC	ACC	Youden	Sen	Spe	PPV	NPV
N	0.696	[0.385-1.000]	0.564	0.713	0.903	0.571	1.000	0.571	0.889	1.000
M	0.589	[0.329-0.849]	0.171	0.345	0.807	0.244	0.958	0.286	0.821	0.667
**NM**	0.756	[0.482-1.000]	0.263	0.713	0.903	0.571	1.000	0.571	0.889	1.000

N, the model with the features only from nodule region; M, the model with the features only from mammary region; NM, the model combining the features from nodule and mammary regions; AUC, area under the curve; CI, confidence interval; MCC, Matthews correlation coefficient; ACC, accuracy; Sen, sensitivity; Spe, specificity; PPV, positive predictive value; NPV, negative predictive value.

## Discussion

In the present study, we developed a radiomics-based model for diagnosis of benign and malignant lesions in the patients with breast cancer. Our results showed that the model combining the features from nodule and mammary regions outperform among all the models, and suggest that the complementary information in mammary region can increase the precision of diagnosis.

DCE-MRI can exhibit blood supply characteristics of the lesions via observing the signal changes among multi-phases (13). The features from enhanced images were closely associated with hemodynamic characteristics, including density and distribution of micro-vessels, vascular proliferation or neovascularization around the lesions (14). Tekpli et al, proved the advantage of DCE-MRI in noninvasively assessing the degree of hypoxia and neovascularization in the tumor microenvironment of breast cancer patients (15). Additionally, it has been validated as a sensitive imaging technique for precise diagnosis of breast lesions and a potential surrogate to pathological biopsy (16). Yu et al. developed and validated the performance of radiomics-based signature in predicting axillary lymph node metastasis and disease-free survival in patients (8). Ya et al. assessed the impact of difference in parameters at different time points in predicting value oof lymph node metastasis of breast cancer (17). The invisible higher-order features on DCE-MRI contributed to precise diagnosis and prediction of treatment.

The progression of breast cancer not only has the effect of tumor cells, but also includes the microenvironment that can promote tumor development (18). Breast cancer invades surrounding tissues during progression, leading to remodeling of the peritumoral structure (19). The peritumoral area composed of parenchymal tissue around the tumor can be considered as the representative of the tumor microenvironment, the subtle changes of which can be detect by DCE-MRI. The radiomics features which reflected the changes of peritumoral environment can thus be used as the indicators for the density of infiltrating lymphocytes around the tumor (20–22). As researchers found the defects in the radiomics research that only extracted features from primary tumor, the role of peritumoral region attracted more attention due to its association with tumor infiltration, vascular proliferation and lymphovascular invasion (12, 23–26). The increase of peritumoral interstitial fibrosis is related to the high invasiveness of the tumor (27), and the presence of peritumoral lymphovascular invasion (LVI) is closely associated with higher distant metastasis rate and mortality (28). Our results also suggested that the model combining the features from the nodule and mammary regions performed best compared with the model using the features only from the nodule region.

However, the distance between peritumoral tissue and the lesion was various, leading to a challenge in clinical application (29, 30). Shin et al, found the difference of proximal, middle and distal peritumoral stroma in differentiating between low-risk and high-risk breast cancer (29) on apparent diffusion coefficient images. Capillary proliferation and neovascularization were a principal manifestation in the patients with breast cancer (31). The features from peritumoral region cannot reflect the wide distribution of abnormal blood vessel growth. There was no exact threshold to classify lesion and peritumoral region. Guo et al, also found that the difference in distance of peritumoral region can impact the performance of AI model in distinguishing malignant from benign nodules (32). Consequently, we extracted the features from mammary regions in the present study, avoiding the uncertainty caused by various definition of tumoral region.

This study had several limitations. First, this study is a single-center study with a small sample size. Future work aimed to test the model on a multi-center dataset. Secondly, only one sequence was used to extract features. Diffusion-weighted imaging and pharmacokinetic images also had predictive value in diagnosis of benign and malignant nodules. Finally, the impact of the features from mammary region was still unclear on predicting molecular subtype, treatment response and overall survival. More studies would be conducted to dig out the value of the features from mammary region in prevention and treatment of breast cancer.

## Conclusion

In conclusion, we applied a radiomics-based method to diagnose nodules on DCE-MRI. Compared with the models only using the features from nodule or mammary region, the model combining the features from nodule and mammary region outperformed in diagnosis of benign and malignant nodules.

## Data availability statement

The raw data supporting the conclusions of this article will be made available by the authors, without undue reservation.

## Ethics statement

Ethical approval was not required for the study involving humans in accordance with the local legislation and institutional requirements. Written informed consent to participate in this study was not required from the participants or the participants’ legal guardians/next of kin in accordance with the national legislation and the institutional requirements.

## Author contributions

WF: Writing – original draft. WS: Writing – original draft. MX: Writing – review & editing. JP: Writing – review & editing. FM: Writing – original draft, Writing – review & editing.
